# Wu-Mei-Wan Reduces Insulin Resistance via Inhibition of NLRP3 Inflammasome Activation in HepG2 Cells

**DOI:** 10.1155/2017/7283241

**Published:** 2017-08-27

**Authors:** Xueping Yang, Lingli Li, Ke Fang, Ruolan Dong, Jingbin Li, Yan Zhao, Hui Dong, Ping Yi, Zhaoyi Huang, Guang Chen, Fuer Lu

**Affiliations:** ^1^Institute of Integrative Traditional Chinese & Western Medicine, Tongji Hospital, Tongji Medical College, Huazhong University of Science & Technology, Wuhan 430030, China; ^2^Department of Traditional Chinese Medicine, Puai Hospital, Tongji Medical College, Huazhong University of Science & Technology, Wuhan 430033, China; ^3^Department of Integrative Traditional Chinese & Western Medicine, Tongji Hospital, Tongji Medical College, Huazhong University of Science & Technology, Wuhan 430030, China

## Abstract

Wu-Mei-Wan (WMW) is a Chinese herbal formula used to treat type 2 diabetes. In this study, we aimed to explore the effects and mechanisms of WMW on insulin resistance in HepG2 cells. HepG2 cells were pretreated with palmitate (0.25 mM) to impair the insulin signaling pathway. Then, they were treated with different doses of WMW-containing medicated serum and stimulated with 100 nM insulin. Results showed that palmitate could reduce the glucose consumption rate in HepG2 cells and impair insulin signaling related to phosphorylation of insulin receptor (IR) and insulin receptor substrate-1 (IRS-1), thereby regulating the downstream signaling pathways. However, medicated serum of WMW restored impaired insulin signaling, upregulated the expression of phospho-IR (pIR), phosphatidylinositol 3-kinase p85 subunit, phosphoprotein kinase B, and glucose transporter 4, and decreased IRS serine phosphorylation. In addition, it decreased the expression of interleukin-1*β* and tumor necrosis factor-*α*, which are the key proinflammatory cytokines involved in insulin resistance; besides, it reduced the expression of NLRP3 inflammasome. These results suggested that WMW could alleviate palmitate-induced insulin resistance in HepG2 cells via inhibition of NLRP3 inflammasome and reduction of proinflammatory cytokine production.

## 1. Introduction

Diabetes has become one of the most important metabolic diseases worldwide and a serious threat to public health. Type 2 diabetes is characterized by impaired insulin secretion and insulin resistance in peripheral tissues [1]. Insulin resistance means that insulin-sensitive tissues, such as the liver, muscles, and adipose tissues, exhibit a lower biological effect when stimulated with the normal serum concentration of insulin. Therefore, the physiological functions of insulin, such as inhibition of glycogen production, promotion of glucose uptake by the muscles and adipose tissues, and inhibition of lipolysis, are affected, resulting in hyperglycemia and hyperlipidemia [2]. Therefore, insulin resistance is a common pathophysiological process in various diseases, including hypertension, obesity, dyslipidemia, and type 2 diabetes [3, 4]. Thus, reducing insulin resistance is of great importance in the prevention and treatment of various metabolic diseases.

Numerous studies have shown that insulin resistance is attributed to chronic nonspecific inflammation, which is mediated by the nonspecific innate immune response, and involves various inflammatory cytokines. Secretion of inflammatory cytokines is a chronic process, which may be the main pathogenic factor leading to insulin resistance [5]. Among the inflammatory cytokines, interleukin-1*β* (IL-1*β*) is believed to be mainly involved in insulin resistance, where it can activate nuclear factor kappa B (NF-*κ*B) and promote the secretion of many other inflammatory factors [6]. Jager et al. confirmed that IL-1*β* impaired insulin signaling by reducing insulin receptor substrate (IRS) activation and suppressing tyrosine phosphorylation of IRS [7].

Previous studies have shown that IL-1*β* is mainly regulated by inflammasomes [8]. As a member of the Nod-like receptors (NLRs), NLRP3 is an important pattern recognition receptor involved in the innate immunity. After interaction with apoptosis-associated speck-like protein containing a caspase recruitment domain (ASC), the activated NLRP3 can recruit caspase-1 precursors and form a protein complex “inflammasome.” Activated NLRP3 inflammasome, in the form of caspase-1 shear body (p20), cleaves the precursor of IL-1*β* to form mature IL-1*β*. Wen et al. showed that free fatty acids, such as palmitate, could induce reactive oxygen species production and RNA release from the mitochondria, activate the NLRP3 inflammasome, and eventually impair insulin signaling [9]. Therefore, inhibition of NLRP3-induced inflammatory response and reduction of insulin resistance are of great significance in the prevention and treatment of type 2 diabetes.

Diabetes has been described in China very long ago, and ancient Chinese doctors suggested various treatment prescriptions, which have proven effective in the clinical treatment of diabetes nowadays. Wu-Mei-Wan (WMW) is one of these prescriptions, which was archived in the classic Shang Han Za Bing Lun (200–210 AD). This formula is composed of ten herbs:* Fructus Mume*,* Herba Asari*,* Rhizoma Zingiberis*,* Rhizoma Coptidis*,* Radix Angelicae sinensis*,* Rhizoma Typhonii gigantei*,* Pericarpium Zanthoxyli*,* Ramulus cinnamomi*,* Radix Ginseng*, and* Cortex Phellodendri*. Our previous study showed that WMW reduced insulin resistance in diabetic rats [10]; however, the underlying mechanism was still unclear. Therefore, we conducted further studies on WMW at the cellular level using HepG2 cells to investigate the underlying mechanisms of its antidiabetic effects.

## 2. Materials and Methods

### 2.1. Preparation of Herbal Decoctions and Medicated Serum and Experimental Design

Components of WMW were purchased from Chinese Herbal Medicine Co., Ltd. (Hubei, China). WMW decoction was prepared in the Institute of Integrative Traditional Chinese and Western Medicine, Tongji Hospital, Tongji Medical College, Huazhong University of Science and Technology (HUST, Hubei, China). Briefly,* Fructus Mume* was soaked in vinegar overnight.* Rhizoma Typhonii gigantei* was preboiled for 2 h, whereas the other herbs were soaked in water for 1 h. The herb mixture was then boiled for 2 h. Another round of boiling for one hour was performed before filtration, and the product was stored at 4°C until use.

Sixteen clean, healthy male Sprague-Dawley rats, weighing 200 ± 20 g, were purchased from Hubei Provincial Center for Disease Control and Prevention and housed in a specific pathogen-free (SPF) room under controlled conditions of temperature (22 ± 2°C), humidity (55 ± 5%), and 12/12 h light/dark cycles. Water and food were given ad libitum. Animal experiments were approved by the animal ethics committee of HUST. Rats were treated intragastrically with WMW at doses of 4.5, 9, and 18 g/kg/day (*n* = 4 each). The control group received sterile water intragastrically (*n* = 4). Treatments were administered at 8:00 am and 8:00 pm daily for 4 consecutive days. Abdominal aortic blood samples were withdrawn under sterile conditions 1 h after the last dose. The blood samples were aseptically packed and centrifuged at 12,000 ×g for 15 min. Then, the serum was inactivated using a water bath at 56°C for 30 min, sterilized using a 0.22 *μ*m filter membrane, and stored at −80°C.

### 2.2. Cell Culture and Treatment

HepG2 cells, provided by the Department of Immunology, Tongji Medical College, HUST, were grown in Dulbecco's modified Eagle's medium (DMEM, HyClone™) supplemented with 10% fetal bovine serum (FBS, Gibco, USA), 100 U/mL penicillin, and 100 *μ*g/mL streptomycin (Solarbio, Beijing, China) and maintained at 37°C under 5% CO_2_. HepG2 cells were seeded into 6-well plates at a density of 3 × 10^5^ cells/well. After 6 h of starvation, HepG2 cells in the logarithmic phase were treated with 0.25 mM palmitate (Sigma, USA) for 24 h as the disease group. Cells in other groups were also treated with three different concentrations of WMW-containing medicated serum besides palmitate pretreatment and stimulated with 100 nM insulin (Sigma, USA) for 15 min or 5 *μ*M nigericin (Sigma, USA) for 45 min. The supernatants and underlying cells were collected for assays.

### 2.3. 3-(4,5-Dimethylthiazol-2-yl)-2,5-diphenyltetrazolium Bromide (MTT) Assay

HepG2 cells were seeded into 96-well plates at a density of 1 × 10^4^ cells/well in DMEM supplemented with 10% FBS (six replicates). After treatment with 0.25 mM palmitate and medicated serum of WMW, cell viability was measured using the MTT reagent (Solarbio, Beijing, China) dissolved in phosphate-buffered saline (PBS, 5 mg/mL). On the day of measurement, the medium was carefully replaced by fresh DMEM containing 10% FBS and diluted MTT (1 : 10, 10% MTT) and incubated for 4 h at 37°C. After removal of the incubation medium, the cells were incubated in 100 *μ*L of dimethylsulfoxide for 30 min. The optical density (OD) was measured at 570 nm using a microplate reader (Synergr 2, BioTek). Relative cell viability was determined as the OD of the treated group divided by the OD of vehicle control.

### 2.4. Glucose Consumption

HepG2 cells were seeded into 96-well plates at a density of 1 × 10^4^ cells/well in DMEM supplemented with 10% FBS (six replicates). Then, cells in the disease group were treated with 0.25 mM palmitate for 12, 24, and 48 h. After stimulation with 100 nM insulin for 15 min, the medium was removed, and glucose concentration was determined by the glucose oxidase method according to the manufacturer's protocol (Nanjing Jiancheng, Nanjing, China). Glucose concentration was calculated by subtracting the glucose concentration in the blank wells from that in the cell-containing wells.

### 2.5. Enzyme-Linked Immunosorbent Assay (ELISA)

ELISA kits of IL-1*β*, monocyte chemoattractant protein-1*α* (MCP-1*α*), and tumor necrosis factor-*α* (TNF-*α*) were purchased from Neobioscience Biological Technology Co., Ltd., China. IL-1*β*, MCP-1*α*, and TNF-*α* concentrations were determined in the supernatants of HepG2 cells according to the manufacturer's instructions.

### 2.6. Antibodies and Chemicals

Antibodies against IL-1*β*, ASC (Novusbio, USA), NLRP3 (R&D, USA), caspase-1 (Santa Cruz, USA), insulin receptor (IR; Abcam, UK), IRS-1 (upstate, USA), phospho-IRS-1 (p-IRS-1) (Ser307), phospho-IR (pIR), phosphoprotein kinase B (pPKB/p-AKT), PKB/AKT, phosphatidylinositol 3-kinase (PI3K) (p85), glucose transporter 4 (GLUT4; Cell Signaling Technology [CST], USA), *β*-actin, and *β*-tubulin (Wuhan Goodbio Technology Co., Ltd., China) were used in this study. Sodium dodecyl sulfate-polyacrylamide gel electrophoresis (SDS-PAGE) components, radioimmunoprecipitation (RIPA) buffer, phenylmethanesulfonyl fluoride (PMSF), and cocktail protease inhibitor were purchased from Wuhan Goodbio Technology Co., Ltd., China. Antifade mounting medium and 4′,6-diamidino-2-phenylindole (DAPI) were purchased from Biosscii Technology Co., Ltd. (China). Bicinchoninic acid (BCA) protein assay reagent was purchased from Bioyear Biological Technology Co., Ltd. (China). Trizol, PrimeScript™ RT Master Mix, and SYBR® Premix Ex Taq™ were purchased from Takara (Japan).

### 2.7. Quantitative Real-Time Polymerase Chain Reaction (RT-qPCR)

Total RNA was extracted using Trizol reagent. The purity and concentration of total RNA were measured by a nucleic acid analyzer (Thermo, Rockford, IL, USA). Then, total RNA (2 *μ*g) was reverse-transcribed. RT-qPCR was performed using an Applied Biosystems StepOne™ Real-Time PCR system (StepOne, Foster City, CA, USA). The mRNA levels of the target genes were normalized to that of glyceraldehyde 3-phosphate dehydrogenase (GAPDH) in the same sample. The primer sequences used in RT-qPCR assays are shown in Table 1.

### 2.8. Western Blot

Proteins in HepG2 cells were extracted by RIPA buffer containing PMSF and cocktail protease inhibitor, and the cell debris was removed by centrifugation at 12,000 ×g for 15 min at 4°C. The proteins were separated on 10–12% SDS-PAGE (100 V, 1.5 h) and transferred to 0.22 *μ*m polyvinylidene difluoride (PVDF) membranes (280 A, 1 kDa/min). Membranes were blocked with bovine serum albumin (BSA) powder in ultrapure water for 1 h at room temperature and incubated with the primary antibodies overnight at 4°C. Horseradish peroxidase- (HRP-) conjugated secondary antibody was applied to the membrane and incubated for 1 h at room temperature after three washes with Tris-Buffered Saline and Tween 20 (TBST, 10 min each). Bands were visualized using Odyssey infrared imaging and normalized against *β*-actin/*β*-tubulin using Image Pro Plus software (Media Cybernetics, USA).

### 2.9. Immunofluorescence Staining

Cells were treated with the medicated serum of WMW for 24 h and then fixed in 4% paraformaldehyde for 30 min at room temperature. They were washed with PBS for three times, permeabilized with 0.5% Triton X-100 for 15 min, and blocked with 5% BSA for 30 min. Then, cells were incubated with anti-IL-1*β* (1 : 200) and anti-caspase-1 (p20; 1 : 50) primary antibodies overnight at 4°C. On the next day, a secondary antibody was applied to the cells and incubated for 1 h at room temperature after three washes with PBS. DAPI was used to stain the nucleus. Images were acquired using CellSens standard system. They were analyzed using Image Pro Plus to calculate the average OD.

### 2.10. Statistical Analysis

All results were expressed as the means ± standard deviation (SD) and analyzed using SPSS 19.0 software. One-way analysis of variance (ANOVA) was used to determine the statistical significance. Least Significant Difference (LSD) test was performed under the circumstances of equal variance; otherwise, Dunnett's T3 test was applied. Meanwhile, descriptive and variance homogeneity tests were also followed. *P* < 0.05 was considered statistically significant.

## 3. Results

### 3.1. Effects of Palmitate (0.25 mM) on Glucose Consumption Rate of HepG2 Cells at Different Treatment Times

**Figure 1:**
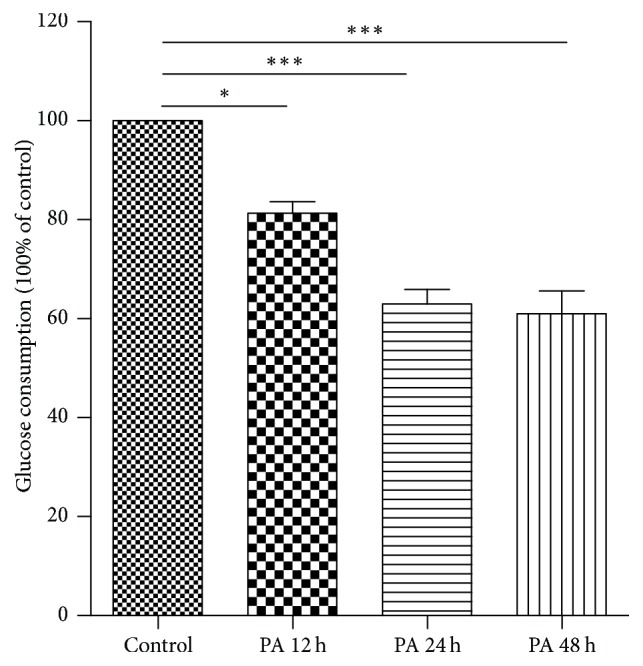
Effects of 0.25 mM palmitate (PA) on glucose consumption rate of HepG2 cells at different treatment times. Control group is HepG2 cells in the normal state without PA treatment, whereas PA 12 h, PA 24 h, and PA 48 h groups are HepG2 cells treated with 0.25 mM palmitate for 12, 24, and 48 h, respectively. Compared to the control group, the glucose consumption rate in palmitate-treated HepG2 cells for 12, 24, and 48 h was 81.3, 63, and 62.3%, respectively. ^*∗*^*P* < 0.05; ^*∗∗∗*^*P* < 0.001.

Insulin resistance is defined as inability of the cells to reach the normal metabolic level under a certain concentration of insulin stimulation, accompanied by deficiency of IR or the components downstream of IR [11]. After 6 h of starvation, HepG2 cells in the logarithmic phase were treated with 0.25 mM palmitate for 12, 24, and 48 h and then stimulated with 100 nM insulin for 15 min. The supernatants were collected to determine the glucose consumption rate. As shown in Figure 1, compared to the control group, the glucose consumption rate of HepG2 cells treated with palmitate for 12 and 24 h decreased to 81.3 and 63%, respectively. However, a slight decrease in glucose consumption rate was observed in HepG2 cells treated with palmitate for 48 h. Therefore, we concluded that glucose utilization by HepG2 cells became impaired after 24 h of palmitate treatment at 0.25 mM.

### 3.2. Effects of the Medicated Serum of WMW on Cell Proliferation in Palmitate-Treated HepG2 Cells

**Figure 2:**
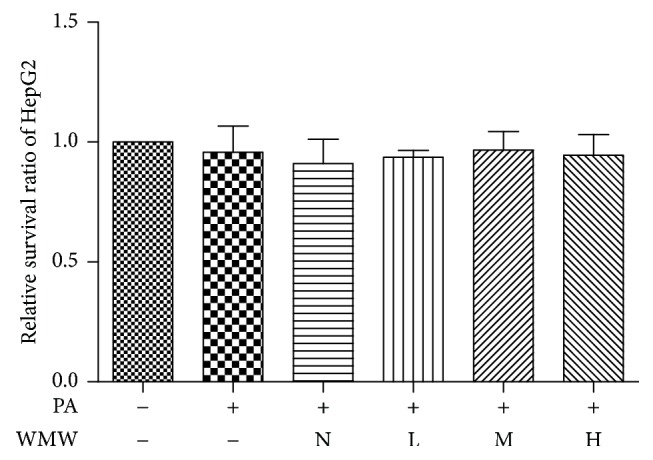
Effects of the medicated serum of WMW on cell proliferation in palmitate-treated HepG2 cells. HepG2 cells were treated with 0.25 mM palmitate for 24 h, and normal serum without WMW (N) or medicated serum containing low (L), middle (M), or high (H) dose of WMW was then added for 24 h. The number of viable cells was examined by the MTT assay.

As shown in Figure 2, there was no significant difference in cell survival rate between the groups treated with the medicated serum of WMW at three different concentrations and the control group. HepG2 cells in normal growth state without palmitate and medicated serum treatment served as the control; the disease group was 0.25 mM palmitate-treated HepG2 cells, whereas the treatment groups consisted of HepG2 cells cotreated with palmitate and the medicated serum of WMW at different doses. Compared to the control group, the survival rate of HepG2 cells in the disease group was 95.8% and that in the normal serum group with no WMW was 91.1%. The survival rate of HepG2 cells in treated with the low, middle, and high doses of WMW medicated serum were 93.7, 96.7, and 95.4%, respectively. There was no significant difference among groups, which indicated that the effects of WMW were independent of cell proliferation.

### 3.3. Effects of WMW on Insulin Signaling Pathway in HepG2 Cells

**Figure 3:**
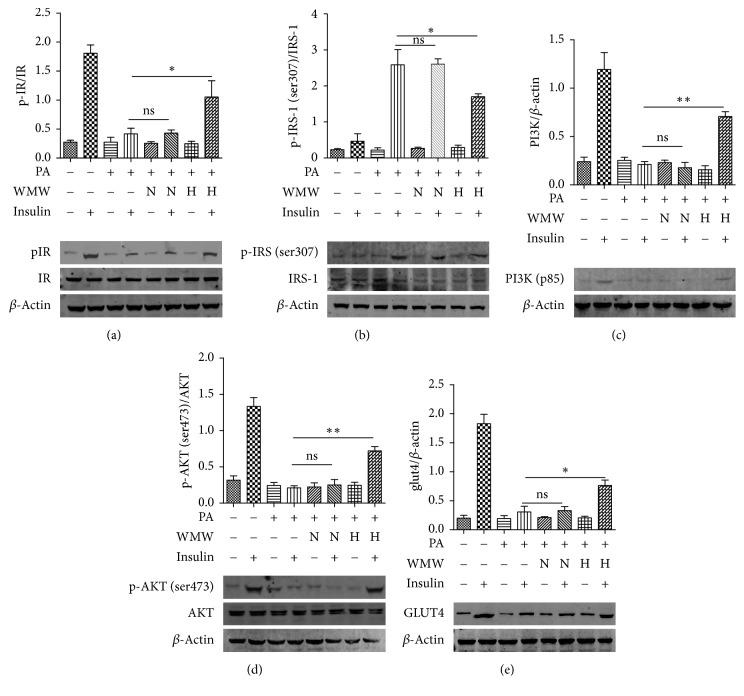
Effects of WMW on insulin signaling pathway in HepG2 cells. HepG2 cells were treated with 10% serum without WMW (N) or medicated serum containing high (H) dose of WMW for 24 h. Then, cells were stimulated with or without 100 nM insulin for 15 min, and cell extracts were collected. The results of western blot analysis of p-IR (a), p-IRS-1 (Ser307) (b), PI3K (p85) (c), p-AKT (d), and GLUT4 (e) are presented by a bar graph. ^*∗*^*P* < 0.05; ^*∗∗*^*P* < 0.01.

After treatment with the medicated serum of WMW, palmitate-treated HepG2 cells were stimulated with 100 nM insulin for 15 min; then, the cells were collected for measurement of the expression of insulin signaling-related proteins. Compared to the control group, the expression of pIR in the palmitate-treated group significantly decreased. However, treatment with the medicated serum of WMW remarkably increased IR phosphorylation (Figure 3(a)). When activated by insulin, IR binds to IRS resulting in its phosphorylation. The phosphorylated IRS combines with the regulatory subunit of PI3K, p85, and activates the downstream signaling pathway. We found that WMW decreased the serine phosphorylation of IRS (Figure 3(b)), which could regulate the expression of downstream PI3K. Expectedly, in our study, we showed that WMW increased PI3K (p85) expression, which could be partly attributed to regulation of IRS phosphorylation (Figure 3(c)).

Downstream of PI3K, AKT is a key molecule in the regulation of insulin signaling pathway. Western blotting was performed to measure the expression of p-AKT and AKT. As shown in Figure 3(d), AKT phosphorylation (ser473) decreased in the disease group, compared to the control group. Treatment with the medicated serum of WMW significantly increased AKT phosphorylation. The expression of GLUT4, which is downstream of p-AKT, was also increased by WMW (Figure 3(e)). Thus, palmitate-induced impairment of insulin signaling in HepG2 cells could be reversed by WMW.

### 3.4. Effects of WMW on Proinflammatory Cytokine Secretion in HepG2 Cells

**Figure 4:**
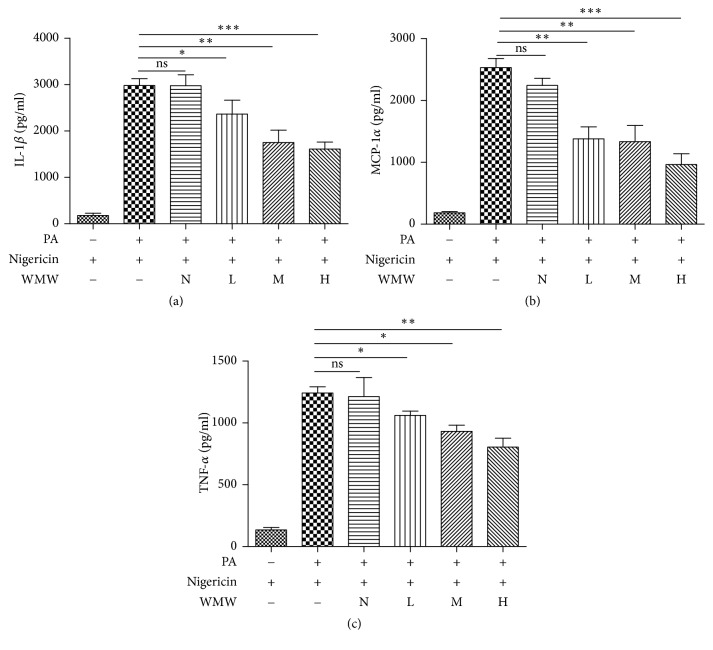
Effects of WMW on the secretion of proinflammatory cytokines in HepG2 cells. HepG2 cells were treated with serum without WMW (N) or medicated serum containing low (L), middle (M), or high (H) dose of WMW for 24 h. Then, cells were stimulated with 5 *μ*M nigericin for 45 min, and the supernatants were collected and analyzed by ELISA for measurement of IL-1*β* (a), MCP-1*α* (b), and TNF-*α* (c) concentrations. ^*∗*^*P* < 0.05, ^*∗∗*^*P* < 0.01, and ^*∗∗∗*^*P* < 0.001.

It has been shown that insulin resistance involves a chronic nonspecific inflammatory reaction. ELISA showed that IL-1*β* levels significantly increased in the disease group, compared to the control group. After treatment with WMW at different doses, IL-1*β* expression decreased (Figure 4(a)). In addition, the medicated serum of WMW reduced the expression of MCP-1*α* (Figure 4(b)) and TNF-*α* (Figure 4(c)) in a dose-dependent manner.

### 3.5. Effects of WMW on mRNA Expression of NLRP3 Inflammasome in HepG2 Cells

**Figure 5:**
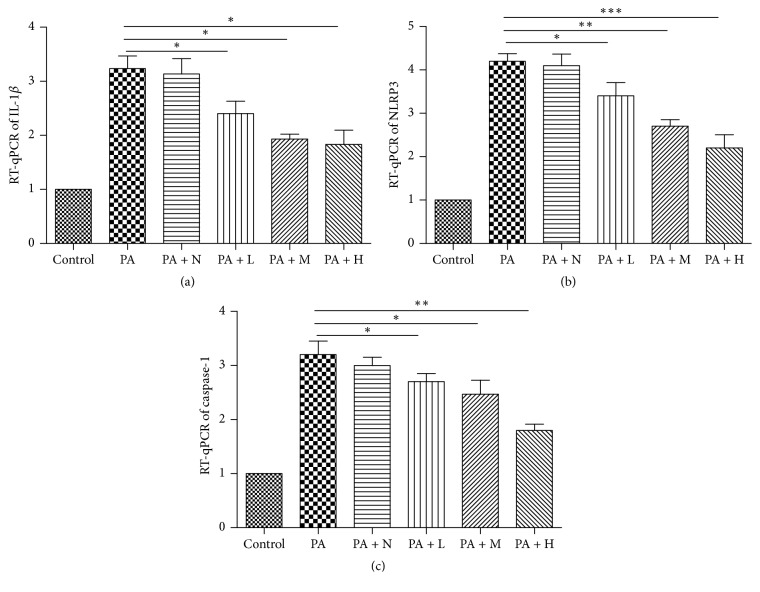
Effects of WMW on mRNA expression of NLRP3 inflammasome in HepG2 cells. Control group is HepG2 cells in the normal state with no palmitate (PA) treatment. HepG2 cells were treated with serum without WMW (PA + N) or medicated serum containing low (PA + L), middle (PA + M), or high (PA + H) dose of WMW for 24 h. RT-qPCR results showed that the medicated serum of WMW increased the mRNA expression of IL-1*β* (a), NLRP3 (b), and caspase-1 (c). ^*∗*^*P* < 0.05, ^*∗∗*^*P* < 0.01, and ^*∗∗∗*^*P* < 0.001.

IL-1*β* is an important inflammatory mediator, which is mainly regulated by NLRP3 inflammasome. We investigated the effects of WMW treatment on the mRNA expression of NLRP3 inflammasome pretreated with palmitate. Consistent with ELISA results, the medicated serum of WMW decreased the mRNA expression of IL-1*β* in HepG2 cells (Figure 5(a)). Furthermore, the mRNA expression of NLRP3 (Figure 5(b)) and caspase-1 (Figure 5(c)) was downregulated by the medicated serum of WMW.

### 3.6. Effects of WMW on the Expression of Proteins Related to NLRP3 Inflammasome Activation in HepG2 Cells

**Figure 6:**
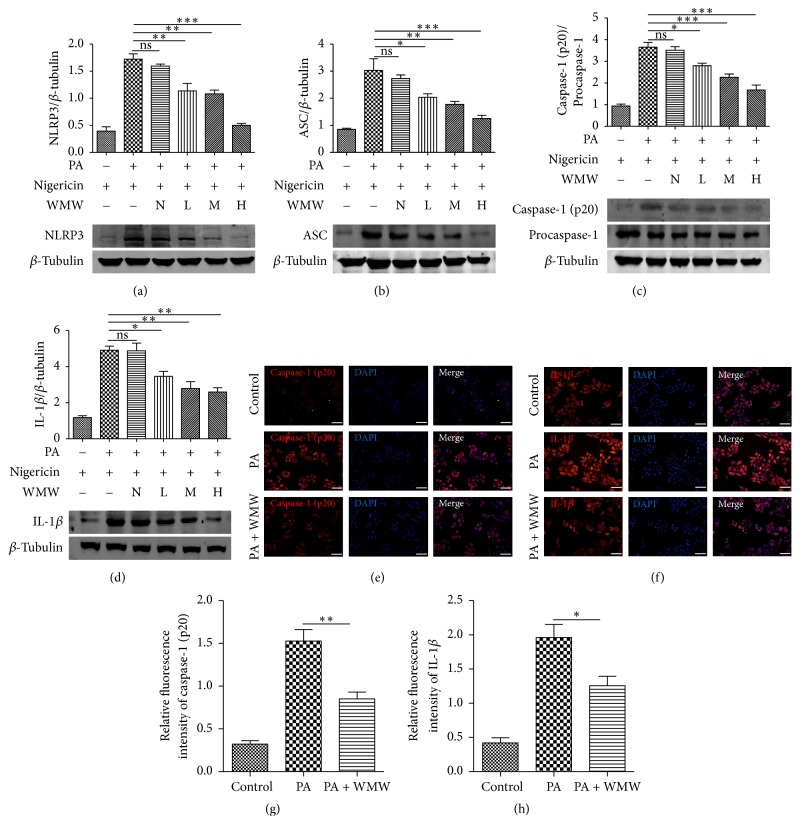
Effects of WMW on the expression of proteins related to NLRP3 inflammasome in HepG2 cells. HepG2 cells were treated with serum without WMW (N) or medicated serum containing low (L), middle (M), or high (H) dose of WMW for 24 h. Then, cells were stimulated with 5 *μ*M nigericin for 45 min, and cell extracts were collected and analyzed by western blotting for measurement of NLRP3 (a), ASC (b), caspase-1 (p20) (c), and IL-1*β* (d) expression. Immunofluorescence staining for caspase-1 (p20) (red) and IL-1*β* (red) in HepG2 cells from the control group, disease group (PA), and group cotreated with serum containing high dose of WMW (PA + WMW) is shown in ((e) and (f)). The nuclei were counterstained with DAPI (blue). Signals for caspase-1 (P20) and IL-1*β* are presented in a bar graph ((g) and (h)). Scale, 100 *μ*m. ^*∗*^*P* < 0.05, ^*∗∗*^*P* < 0.01, and ^*∗∗∗*^*P* < 0.001.

Western blotting and immunofluorescence staining were then performed to confirm the inhibitory effects of WMW on NLRP3 inflammasome activation. We found that the expression of NLRP3 (Figure 6(a)) and ASC (Figure 6(b)) proteins increased in the disease group, compared to the control group. There was no significant difference between the control group and the normal serum-treated group, whereas the medicated serum of WMW decreased the expression of NLRP3 and ASC, which indicates that WMW, not the serum, is responsible for these effects. A previous study revealed that after binding to ASC, NLRP3 recruited procaspase-1 and activated it into caspase-1 (p20). We showed that the medicated serum of WMW decreased the expression of caspase-1 (p20) (Figure 6(c)), which might indicate that WMW interferes with the recruitment of pro-IL-1*β* resulting in less production of IL-1*β* (Figure 6(d)). This, in turn, would inhibit the inflammatory reaction and reduce insulin resistance.

In consistency with the results of western blotting, the inhibitory effects of WMW on the expression of inflammasome-related proteins (caspase-1 [p20] and IL-1*β*) were confirmed by immunofluorescence staining. As shown in Figures 6(e)-6(f), compared to the control group, palmitate increased the expression of caspase-1 (p20) and IL-1*β*; however, high-dose WMW decreased their expression. The fluorescence intensity of caspase-1 (p20) and IL-1*β* staining in different groups is showed in Figures 6(g)-6(h).

## 4. Discussion

WMW, an ancient prescription used for the treatment of diabetes, has been widely used in modern medicine.* Rhizoma Coptidis* [12],* Radix Ginseng* [13], and* Radix Angelicae sinensis* [14], three components of WMW, have been proven to attenuate insulin resistance. In addition, Lu et al. found that WMW could reduce the fasting blood glucose level in diabetic mice [15], and Tu et al. reported similar results in a randomized controlled clinical trial [16]. However, the underlying mechanism remains to be elucidated. Our previous study showed that WMW could regulate IR expression, IRS-1 activation, and GLUT4 expression in the liver, skeletal muscles, and adipose tissues of diabetic rats, which improved insulin sensitivity in peripheral tissue [10]. Therefore, this study further explored the effects and mechanisms of WMW on the insulin signaling pathway at the cellular level. Consistent with the results of our previous study, WMW repaired the impaired insulin signaling induced by palmitate and alleviated insulin resistance.

Much evidence suggested that excessive accumulation of free fatty acids in the serum may be the most primitive cause of insulin resistance [17, 18]. Palmitic acid, which accounts for 30–35% of the total free fatty acids, has been shown to exhibit a direct effect on the insulin signaling pathway in hepatocytes [19]. Our study showed that palmitate could reduce the glucose consumption rate of HepG2 cells and affect insulin signaling related to phosphorylation of IR and IRS-1, whereas WMW relieved these deficits. This study found that the medicated serum of WMW increased IRS-1 phosphorylation in HepG2 cells stimulated by a certain concentration of insulin. Once IRS is activated, its serine phosphorylation decreases. This increases the expression of PI3K and activates AKT, which is crucial for glucose uptake and glycogen synthesis [20], and thus increases the expression of GLUT4.

Insulin resistance is a chronic inflammatory reaction, which involves the accumulation of various inflammatory factors, such as IL-1*β*. Binding of IL-1*β* to its receptor can activate interleukin-1 receptor-associated kinase 1 (IRAK-1). IRAK-1 activation leads to activation of C-JUN terminal kinase and phosphorylation of IRS (Ser307) [21]. Thus, when the interaction of IR with insulin is suppressed, tyrosine phosphorylation of the receptor is inhibited. This also leads to degradation of IRS-1 and inhibits insulin signaling [22]. Fujishiro et al. revealed that IL-1*β* activated p38 mitogen-activated protein kinase [23], which affected the transmembrane transport of glucose. It was shown that IL-1 receptor antagonists reduced the inflammatory reaction and improved insulin resistance in clinical trials [24]. In addition, Stienstra et al. showed that IL-1*β* knockout mice had better insulin sensitivity than wild-type mice [25], which is consistent with the findings of our study which showed that WMW improved insulin sensitivity via reduction of IL-1*β* secretion in HepG2 cells. TNF-*α* is another important inflammatory factor. Hotamisligil et al. showed that TNF-*α* could promote serine phosphorylation of IRS-1 and play an important role in obesity-induced insulin resistance [26]. In addition, TNF-*α* can promote the secretion of MCP-1, which further induces the secretion of many proinflammatory cytokines that can aggravate insulin resistance through various mechanisms [27]. This study showed that palmitate-induced secretion of TNF-*α* and MCP-1*α* in HepG2 cells decreased in the cells treated with the medicated serum of WMW. In addition, in metabolic stress conditions, such as hyperglycemia and hyperlipidemia, NLRP3 combines with ASC and procaspase-1 to form activated NLRP3 inflammasome. This process requires two signals [28]; the first signal is NF-*κ*B activation by many intracellular and extracellular molecules, which upregulates the mRNA expression of NLRP3 and IL-1*β* precursor; additionally, NF-*κ*B can be regulated by TNF-*α* through the IKK kinase complex [29]. Therefore, TNF-*α* is also involved in the activation of NLRP3 inflammasome. Chow et al. revealed that TNF-*α* could regulate NLRP3 inflammasome [30]. Our study showed that WMW downregulated the mRNA expression of NLRP3 in HepG2 cells.

Palmitate can cause insulin resistance by inducing mitochondrial reactive oxygen species release [31] and endoplasmic reticulum stress [32]; however, the involved signaling pathways have not been fully revealed. Reactive oxygen species act as the second signal required for activation of NLRP3 inflammasome [33]. Li et al. revealed that palmitate induced insulin resistance through endoplasmic reticulum stress-mediated NLRP3 inflammasome activation [34]. Thus, NLRP3 inflammasome plays an important role in palmitate-induced insulin resistance. Vandanmagsar et al. also showed that NLRP3 could identify uric acid, ATP, fatty acids, and other endogenous signals; in addition, it might be activated by a lysosome-dependent pathway, which resulted in increased expression of IL-1*β* and other proinflammatory cytokines in the macrophages and induced insulin resistance in the adipose tissues, muscles, liver, and other peripheral tissues [35]. NLRP3 knockout mice were protected against insulin resistance induced by high-fat diet [36]. Our study showed that WMW inhibited IL-1*β* production and relieved insulin resistance in palmitate-treated HepG2 cells via reduction of the expression of the three components of NLRP3 inflammasome: NLRP3, ASC, and caspase-1 (p20).

Therefore, this study showed that WMW could reduce the production of proinflammatory cytokines and alleviate palmitate-induced insulin resistance via inhibition of NLRP3 inflammasome activation in HepG2 cells. The findings of this study could help to uncover the specific mechanisms underlying the hypoglycemic effects of WMW; in addition, it provides evidence that WMW could be used in clinical practice to treat type 2 diabetes and other metabolic inflammatory diseases.

## Figures and Tables

**Table 1:** Real-time PCR primer sequences.

Gene	Forward (5′ → 3′)	Reverse (3′ → 5′)
GAPDH (human)	ACTTTGGTATCGTGGAAGGACTCAT	GTTTTTCTAGACGGCAGGTCAGG
NLRP3 (human)	GATCTTCGCTGCGATCAACAG	CGTGCATTATCTGAACCCCAC
Caspase-1 (human)	TTTCCGCAAGGTTCGATTTTCA	GGCATCTGCGCTCTACCATC
IL-1*β* (human)	CGATCACTGAACTGCACGCTC	ACAAAGGACATGGAGAACACCACTT

## Abbreviations

ASC: Apoptosis-associated speck-like protein containing a CARD domain
ATP: Adenosine-triphosphate triphosphate
Caspase-1: Cysteinyl aspartate specific proteinase 1
DAPI: 4′,6-Diamidino-2-phenylindole
ELISA: Enzyme-linked immunosorbent assay
FBS: Fatal bovine serum
GAPDH: Glyceraldehyde 3-phosphate dehydrogenase
GLUT4: Glucose transporter 4
IL-1*β*: Interleukin-1*β*
IRAK-1: Interleukin-1 receptor-associated kinase 1
IR: Insulin receptor
IRS: Insulin receptor substrate
MCP-1*α*: Monocyte chemoattractant protein-1*α*
MTT: 3-(4,5-Dimethylthiazol-2-yl)-2,5-diphenyltetrazolium bromide
NF-*κ*B: Nuclear factor-*κ*-gene binding
NLRP3: Nucleotide-binding oligomerization domain, leucine rich repeat, and pyrin domain containing 3
NLRs: Leucine rich repeat-containing receptor
NOD: Nucleotide combining oligomerization domain
PBS: Phosphate buffer saline
PKB/AKT: Protein kinase B
PI3K: Phosphatidylinositol 3-kinase
pIR: Phosphoinsulin receptor
PMSF: Phenylmethanesulfonyl fluoride
pPKB/p-AKT: Phosphoprotein kinase B
Pro-IL-1*β*: Precursor of interleukin-1*β*
RIPA: Radioimmunoprecipitation
RT-qPCR: Quantitative real-time polymerase reaction analysis
SPF: Specific pathogen-free
TBST: Tris-Buffered-Saline and Tween 20
WMW: Wu-Mei-Wan
TNF-*α*: Tumor necrosis factor-*α*.
